# Monthly Summary

**Published:** 1883-04

**Authors:** 


### MONTHLY SUMMARY.

---

*Teeth Injured by Tobacco.*—" I was taught that the use of tobacco in any form was not injurious to the teeth, and in all the literature of the profession I have found nothing alluding to what I desire to present to the profession; namely, the evil effects upon the teeth, caused by the constant use of tabacco. My attention was first drawn to this evil just one year ago this month, when I was filling the teeth of a patient who has for years been in the habit of smoking and chewing a great deal of tobacco.

" The injurious effects are not very noticable until the person has been using the weed for about fifteen years, but the use of the pipe to excess will show its injurious effects in less time. Tobacco chewing is the most injurious, as the tobacco acts as an irritant in two ways, mechanically and by its properties—mechanically by particles of the tobacco being forced between the gums and the teeth We have proofs of the irritable effects of tobacco in snuff. The direct effect of using tobacco is the recession ot the gums of all the teeth, but more especially those on the side of the mouth used most in chewing the tobacco. The sequel to this recession may cause the loss of one or more teeth, by a diseased condition of the pulp, resulting from its being irritated by having the neck of the tooth and the root exposed to thermal changes in food and in the air we breathe. Exostosis and calcification may result.

" Tobacco chewers' teeth wear away on the grinding surface rapidly, caused by the gritty substances naturally entering into the tobacco. The gums recede and are red and congested, and underneath the gum a narrow line of dark tartar is nearly always present, and particles may be found still further toward the apex of the tooth.—*Dr. John G. Harper, in Southern Dental Journal.*

---

*Separation of Silver from Alloys.*—Mr. Solthien describes the following simple method for separating silver from alloys:

The silver-holding alloy or metals are dissolved in the least possible quantity of crude nitric acid. The solution is mixed with a strong excess of ammonia and filtered into a high cylinder, provided with a stopper. A bright strip of copper, long enough to project beyond the liquid, is next introduced, which quickly causes separation of pure metallic silver. The reduction is completed in a short time, and the reduced silver is washed, first with some ammoniacal and then with distilled water.

The more ammoniacal concentrated the solution was, the more rapid is the reduction. The strip of cotton should not be too thin, as it is considerably attacked, and any little particles which might separate from a thin sheet would contaminate the silver.

The operation is so simple that it seems preferable to all others for such operations as the preparation of nitric of silver from old coins. etc. Any accompanying gold remains behind during the treatment of the metal or alloy with nitric acid, chloride of silver (produced by the impurities [H C I] in the nitric acid) is taken by the ammoniacal solution, like the copper, and is also reduced to the metallic state; and whatever other metal was not left behind, oxodized by the nitric acid, is separated as hydrate (as lead, bismuth) on treating with ammonia. Any arsenate which may have passed into the ammoniacal solution is not decomposed by the copper.—*Arch. d. Pharm.*

---

*A Conclusion Not Warranted by Facts.*—The *Independent Practitioner* says that "Mr. William J. Thulman, a druggist of Buffalo, recently came to his death from a singular cause. While eating his dinner a large amalgam filling in one of his teeth became detatched and was swallowed. He immediately expressed his apprehension of trouble from it, but felt no special inconvenience for some days, when he began to experience pain in the abdominal region. The symptoms became aggravated, peritonitis, ensued, and he finally died after much suffering. Autopsy was held by prominent physicians, when it was found that the irregularly shaped mass had lodged in one of the lower folds of the ilium, and had produced an ulcer which had eaten its way through the intestines, and finally caused his death."

One of the "prominent physicians" who was present at the autopsy says that neither an "irregularly shaped mass," or anything that looked like an amalgam filling was found, although diligent search was made. We are inclined to think that the conclusions in the case are not warranted by the facts. —*Dental News.*

---

*Gold Alloy.*—Dr. W. H. Dorrance prepares gold solder after this manner: take of pure silver one part, of zinc two parts and of copper three parts. Melt the silver and copper together and add the zinc slowly, in small pieces, allowing all the fumes to pass off. If, after thorough mixing, this is thrown into water, it will separate into small pellets. With these reduce the gold to the proper degree of fineness. If pure gold is used, the number of parts out of twenty-four which are taken will express the fineness of the solder in carats. Twenty parts of gold will make 20-k. solder; sixteen parts, 16-k. solder, etc. If U. S. coin be used, there will be two parts more of alloy, and twenty parts of gold will make but 18-k. solder. After melting it may be run into an ingot, when it will readily roll out into a plate of any desired thickness.

We have used solder made in this manner, and can express our entire satisfaction with it. It bears an excellent color, flows easily and is very strong. We are under obligations to Dr. Dorrance for this formula, as well as for other courtesies.—*Independent Practitioner.*

---

*Filling Over-Exposed Nerve.*—If the tooth has ached only a little, say two or three times, I rinse out the cavity with warm water; then wipe it with a little water and baking soda. Open the cavity as much as it is to be when filled. Remove the decay all around the margin of the cavity, and some from not near the nerve locality. Leave any decay over the nerve that is not loose enough to wash away. Wipe cavity dry, Then wipe it with creosote. Wipe out the creosote, nearly dry. Now wipe cavity with thick solution gum sandarach. Wipe this varnish away from walls at margin of the cavity. Now fill cavity with water-tight amalgam, packing it all about the pulp location, very gently; the pulp will get covered with the filling without direct pressure. Care needed all the time to avoid pressure of the pulp. In filling margins, draw the amalgam towards the outer edges and let some waste by dropping outside; in this way the varnish at the edges is removed.

Nine cases out of ten such cases remain comfortable after I have treated them.

The soda quiets the pulp by destroying acidity.

The varnish excludes air, and is a non-conductor of heat and electricity.—*H. S. C., in Missouri Dental Journal.*

---

*A Dentist's Wife's Death.*—Mrs. Laura B. Watts, wife of Dr. E. M. Watts, of Portsmuth, expired while under the influence of chloroform, administered by her husband to have her teeth extracted. Atter the operation she spoke a few words and died instantly.—*Local Paper.*

